# Crystal structure of (*E*)-*N*-{[3-methyl-1-phenyl-5-(1*H*-pyrrol-1-yl)-1*H*-pyrazol-4-yl]methyl­idene}hydroxyl­amine

**DOI:** 10.1107/S1600536814023514

**Published:** 2014-10-31

**Authors:** Joel T. Mague, Shaaban K. Mohamed, Mehmet Akkurt, Talaat I. El-Emary, Mustafa R. Albayati

**Affiliations:** a Department of Chemistry, Tulane University, New Orleans, LA 70118, USA; b Chemistry and Environmental Division, Manchester Metropolitan University, Manchester M1 5GD, England; c Chemistry Department, Faculty of Science, Minia University, 61519 El-Minia, Egypt; d Department of Physics, Faculty of Sciences, Erciyes University, 38039 Kayseri, Turkey; e Department of Chemistry, Faculty of Science, Assiut University, 71515 Assiut, Egypt; f Kirkuk University, College of Science, Department of Chemistry, Kirkuk, Iraq

**Keywords:** crystal structure, pyrrole ring, hydrogen bonding, hydroxyl­amine

## Abstract

The title compound, C_15_H_14_N_4_O, crystallizes with two mol­ecules in the asymmetric unit with similar conformations (r.m.s. overlay fit for the 20 non-H atoms = 0.175 Å). In the first mol­ecule, the dihedral angles between the planes of the central pyrazole ring and the pendant phenyl and pyrrole rings are 42.69 (8) and 51.88 (6)°, respectively, with corresponding angles of 54.49 (7) and 49.61 (9)°, respectively, in the second mol­ecule. In the crystal, the two mol­ecules, together with their inversion-symmetry counterparts, are linked into tetra­mers by O—H⋯N hydrogen bonds. The tetra­mers form layers parallel to (211) through pairwise C—H⋯π inter­actions.

## Related literature   

For use of pyrazoles in synthesis of polyfunctionally substituted heterocycles, see: Elnagdi *et al.* (1987 ▶); Quiroga *et al.* (2007 ▶, 2008**a* ▶,b* ▶); Aly *et al.* (1994 ▶). For pharmaceutical properties of pyrazole-containing compounds, see: Bazgir *et al.* (2008 ▶); Dias *et al.* (1994 ▶); El-Kashef *et al.* (2000 ▶); El-Emary *et al.* (2002 ▶).
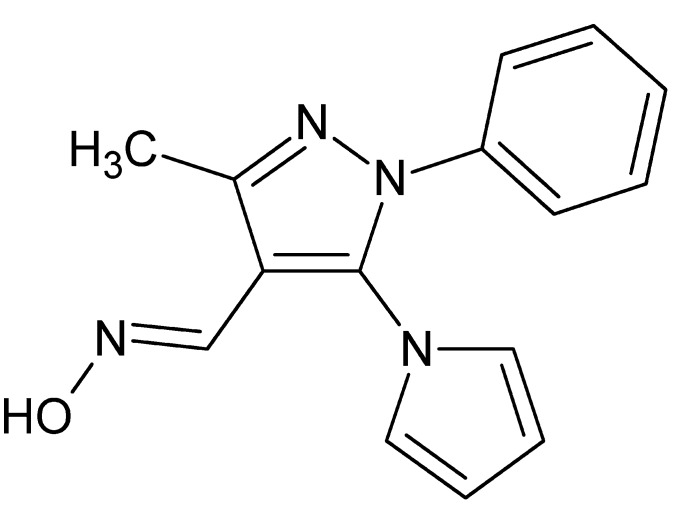


## Experimental   

### Crystal data  

C_15_H_14_N_4_O
*M* *_r_* = 266.30
Triclinic, 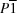

*a* = 9.1497 (2) Å
*b* = 12.3932 (3) Å
*c* = 12.7294 (3) Å
α = 87.4070 (11)°
β = 82.6740 (12)°
γ = 75.0190 (12)°
*V* = 1382.88 (6) Å^3^
*Z* = 4
Cu *K*α radiation
μ = 0.68 mm^−1^
*T* = 150 K
0.22 × 0.15 × 0.05 mm

### Data collection  

Bruker D8 VENTURE PHOTON 100 CMOS diffractometer
Absorption correction: multi-scan (*SADABS*; Bruker, 2014 ▶) *T*_min_ = 0.92, *T*_max_ = 0.97
15570 measured reflections
5370 independent reflections
4088 reflections with *I* > 2σ(*I*)
*R*_int_ = 0.036

### Refinement  

*R*[*F* ^2^ > 2σ(*F* ^2^)] = 0.042
*wR*(*F* ^2^) = 0.108
*S* = 1.04
5370 reflections
363 parameters
H-atom parameters constrained
Δρ_max_ = 0.32 e Å^−3^
Δρ_min_ = −0.19 e Å^−3^

### 

Data collection: *APEX2* (Bruker, 2014 ▶); cell refinement: *SAINT* (Bruker, 2014 ▶); data reduction: *SAINT*; program(s) used to solve structure: *SHELXT* (Sheldrick, 2008 ▶); program(s) used to refine structure: *SHELXL2014* (Sheldrick, 2008 ▶); molecular graphics: *DIAMOND* (Brandenburg & Putz, 2012 ▶); software used to prepare material for publication: *SHELXTL* (Sheldrick, 2008 ▶).

## Supplementary Material

Crystal structure: contains datablock(s) I, global. DOI: 10.1107/S1600536814023514/hb7307sup1.cif

Structure factors: contains datablock(s) I. DOI: 10.1107/S1600536814023514/hb7307Isup2.hkl

Supporting information file. DOI: 10.1107/S1600536814023514/hb7307Isup3.cml

**Fig. 1.**
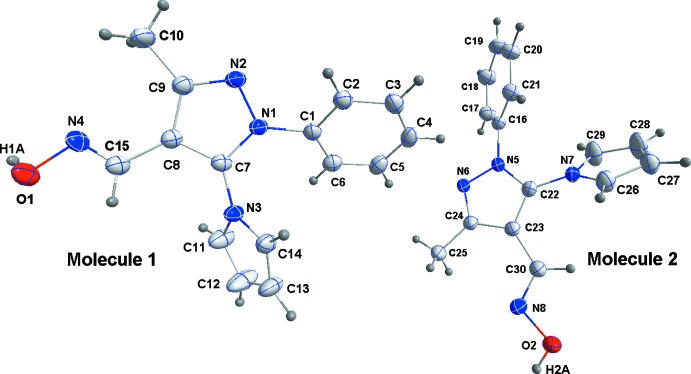
The asymmetric unit of the title compound with 50% probability ellipsoids.

**Fig. 2.**
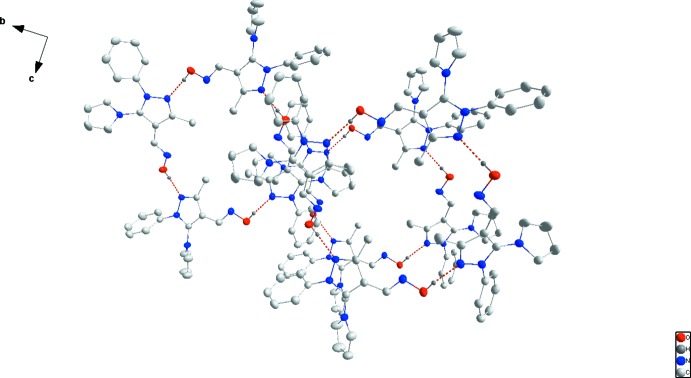
Packing of three H-bonded tetramers viewed down the a axis. O—H···N hydrogen bonds are shown by dotted lines.

. DOI: 10.1107/S1600536814023514/hb7307fig1.tif
The asymmetric unit of the title compound with 50% probability ellipsoids.

a . DOI: 10.1107/S1600536814023514/hb7307fig2.tif
Packing of three H-bonded tetra­mers viewed down the *a* axis. O—H⋯N hydrogen bonds are shown by dotted lines.

**Fig. 3.**
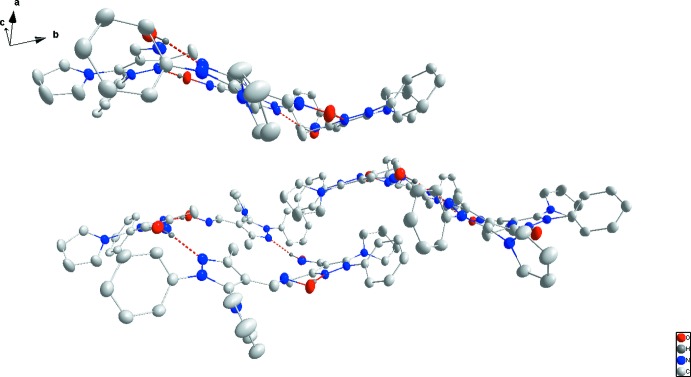
Elevation view of the layers of H-bonded tetramers.

. DOI: 10.1107/S1600536814023514/hb7307fig3.tif
Elevation view of the layers of H-bonded tetra­mers.

CCDC reference: 1031015

Additional supporting information:  crystallographic information; 3D view; checkCIF report

## Figures and Tables

**Table 1:** Hydrogen-bond geometry (, ) *Cg* is centroid of C1C6 ring.

*D*H*A*	*D*H	H*A*	*D* *A*	*D*H*A*
O2H2*A*N2^i^	0.84	1.95	2.7835(19)	174
O1H1*A*N6^ii^	0.84	1.99	2.8277(19)	172
C11H11*Cg* ^ii^	0.95	3.45	?	170

Symmetry codes: (i) 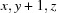; (ii) 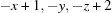.

## supplementary crystallographic information

### S1. Comment

Pyrazoles are interested class of heterocyclic compounds for chemists and pharmacists due to their diverse synthetic and biological applications. Pyrazoles are excellent precursors for the synthesis of condensed polyfunctionally substituted heterocycles (Elnagdi *et al.*, 1987; Quiroga *et al.*, 2007; Quiroga *et al.*, 2008*a,b*; Aly *et al.*, 1994). Moreover, pyrazole containing compounds exhibit a broad spectrum of pharmaceutical properties such as anti-hyperglycemic and analgesic (Bazgir, *et al.*, 2008), anti-parasitic (Dias *et al.*, 1994) anti-microbial (El-Kashef *et al.*, 2000) and anti-schistosomal activities (El-Emary *et al.*, 2002). Following our on-going study in the synthesis and characterization of new bio-active heterocyclic compounds, we report here the crystal structure determination of the title compound.

There are two independent molecules of the title compound in the asymmetric unit which differ primarily in the orientation of the pendant phenyl and pyrrolyl rings. Thus the dihedral angles between these rings, respectively, and the central heterocyclic ring are 42.69 (8) and 51.88 (6)° in molecule 1 but 54.49 (7) and 49.61 (9)° in molecule 2. Molecules 1 at *x*, *y*, *z* and 1 - *x*, -1 - *y*, 2 - *z* and molecules 2 at *x*, -1 + *y*, *z* and 1 - *x*, -*y*, 2 - *z* are associated into a cyclic tetramer *via* O—H···N(2 or 6) hydrogen bonds (Table 1 and Fig. 2). These units form sheets approximately parallel to (100) (Fig. 2) in which the major inter-tetramer interaction within the sheet appears to be pairwise C—H···π (C11—H11···*Cg*: H···*Cg* = 3.45 Å, C—H···*Cg* = 170° (*Cg* is centroid of C1–C6 ring at 1 - *x*, -*y*, 2 - *z*).

### S2. Experimental

A mixture of 760 mg (3 mmol) 3-methyl-1-phenyl-5-(1*H*-pyrrol-1-yl)-4,5-dihydro-1*H*-pyrazole-4-carbaldehyde and 208.5 mg (3 mmol) of hydroxylamine hydrochloride in 15 ml pyridine was heated under reflux for 3 h. After cooling, the reaction mixture was poured into cold water. The resulting solid product was filtered, washed with water, dried under vacuum and crystallized from dioxane to give colourless plates Yield 76%, m.p. 463–465 K.

### S3. Refinement

H-atoms attached to carbon atoms were placed in calculated positions (C—H = 0.95 - 0.98 Å) while those attached to oxygen atoms were placed in locations derived from a difference map and their parameters adjusted to give O—H = 0.84 Å. All were included as riding contributions with isotropic displacement parameters 1.2 - 1.5 times those of the attached atoms.

### Crystal data

**Table:**

C_15_H_14_N_4_O	*Z* = 4
*M**_r_* = 266.30	*F*(000) = 560
Triclinic, *P*1	*D*_x_ = 1.279 Mg m^−^^3^
*a* = 9.1497 (2) Å	Cu *K*α radiation, λ = 1.54178 Å
*b* = 12.3932 (3) Å	Cell parameters from 8765 reflections
*c* = 12.7294 (3) Å	θ = 3.5–72.2°
α = 87.4070 (11)°	µ = 0.68 mm^−^^1^
β = 82.6740 (12)°	*T* = 150 K
γ = 75.0190 (12)°	Plate, colourless
*V* = 1382.88 (6) Å^3^	0.22 × 0.15 × 0.05 mm

### Data collection

**Table:**

Bruker D8 VENTURE PHOTON 100 CMOS diffractometer	5370 independent reflections
Radiation source: INCOATEC IµS micro-focus source	4088 reflections with *I* > 2σ(*I*)
Mirror monochromator	*R*_int_ = 0.036
Detector resolution: 10.4167 pixels mm^-1^	θ_max_ = 72.4°, θ_min_ = 3.5°
ω scans	*h* = −11→11
Absorption correction: multi-scan (*SADABS*; Bruker, 2014)	*k* = −15→15
*T*_min_ = 0.92, *T*_max_ = 0.97	*l* = −15→15
15570 measured reflections	

### Refinement

**Table:**

Refinement on *F*^2^	Primary atom site location: structure-invariant direct methods
Least-squares matrix: full	Secondary atom site location: difference Fourier map
*R*[*F*^2^ > 2σ(*F*^2^)] = 0.042	Hydrogen site location: mixed
*wR*(*F*^2^) = 0.108	H-atom parameters constrained
*S* = 1.04	*w* = 1/[σ^2^(*F*_o_^2^) + (0.0468*P*)^2^ + 0.401*P*] where *P* = (*F*_o_^2^ + 2*F*_c_^2^)/3
5370 reflections	(Δ/σ)_max_ = 0.001
363 parameters	Δρ_max_ = 0.32 e Å^−^^3^
0 restraints	Δρ_min_ = −0.19 e Å^−^^3^

### Special details

**Table:**

Geometry. All e.s.d.'s (except the e.s.d. in the dihedral angle between two l.s. planes)are estimated using the full covariance matrix. The cell e.s.d.'s are takeninto account individually in the estimation of e.s.d.'s in distances, anglesand torsion angles; correlations between e.s.d.'s in cell parameters are onlyused when they are defined by crystal symmetry. An approximate (isotropic)treatment of cell e.s.d.'s is used for estimating e.s.d.'s involving l.s. planes.
Refinement. Refinement of *F*^2^ against ALL reflections. The weighted *R*-factor *wR* andgoodness of fit *S* are based on *F*^2^, conventional *R*-factors *R* are basedon *F*, with *F* set to zero for negative *F*^2^. The threshold expression of*F*^2^ > σ(*F*^2^) is used only for calculating *R*-factors(gt) *etc*. and isnot relevant to the choice of reflections for refinement. *R*-factors basedon *F*^2^ are statistically about twice as large as those based on *F*, and *R*-factors based on ALL data will be even larger. H-atoms attached to carbonwere placed in calculated positions (C—H = 0.95 - 0.98 Å) while thoseattached to oxygen were placed in locations derived from a differencemap and their parameters adjusted to give O—H = 0.84 Å. All wereincluded as riding contributions with isotropic displacementparameters 1.2 - 1.5 times those of the attached atoms.

### Fractional atomic coordinates and isotropic or equivalent isotropic displacement parameters (Å^2^)

**Table:**

	*x*	*y*	*z*	*U*_iso_*/*U*_eq_	
O1	0.01620 (14)	−0.14968 (11)	1.23494 (9)	0.0350 (3)	
H1A	0.0512	−0.2083	1.2691	0.042*	
N1	0.32895 (16)	0.00206 (11)	0.85512 (11)	0.0253 (3)	
N2	0.33432 (17)	−0.10531 (12)	0.83004 (11)	0.0283 (3)	
N3	0.23015 (16)	0.13164 (11)	0.99916 (11)	0.0265 (3)	
N4	0.11882 (17)	−0.16601 (13)	1.14130 (11)	0.0305 (3)	
N5	0.86241 (16)	0.34350 (11)	0.53889 (10)	0.0260 (3)	
C1	0.40366 (18)	0.06699 (13)	0.78205 (13)	0.0252 (3)	
C2	0.39036 (19)	0.06265 (14)	0.67514 (13)	0.0281 (4)	
H2	0.3287	0.0198	0.6516	0.034*	
C3	0.4681 (2)	0.12150 (15)	0.60299 (14)	0.0318 (4)	
H3	0.4610	0.1181	0.5295	0.038*	
C4	0.5558 (2)	0.18502 (15)	0.63761 (15)	0.0332 (4)	
H4	0.6089	0.2251	0.5878	0.040*	
C5	0.5667 (2)	0.19049 (15)	0.74439 (15)	0.0323 (4)	
H5	0.6256	0.2356	0.7678	0.039*	
C6	0.49199 (19)	0.13047 (14)	0.81776 (14)	0.0294 (4)	
H6	0.5011	0.1328	0.8911	0.035*	
C7	0.25257 (19)	0.02755 (14)	0.95310 (13)	0.0248 (3)	
C8	0.20937 (19)	−0.06590 (14)	0.99446 (13)	0.0256 (3)	
C9	0.2641 (2)	−0.14714 (14)	0.91388 (13)	0.0282 (4)	
C10	0.2486 (3)	−0.26404 (15)	0.91273 (16)	0.0423 (5)	
H10A	0.2917	−0.2969	0.8434	0.063*	
H10B	0.1406	−0.2636	0.9260	0.063*	
H10C	0.3034	−0.3084	0.9680	0.063*	
C11	0.2741 (3)	0.14651 (16)	1.09638 (15)	0.0413 (5)	
H11	0.3152	0.0889	1.1443	0.050*	
C12	0.2485 (3)	0.25693 (18)	1.11112 (17)	0.0558 (6)	
H12	0.2681	0.2911	1.1713	0.067*	
C13	0.1869 (3)	0.31322 (15)	1.02105 (16)	0.0416 (5)	
H13	0.1580	0.3918	1.0101	0.050*	
C14	0.1765 (2)	0.23523 (14)	0.95337 (14)	0.0295 (4)	
H14	0.1392	0.2492	0.8865	0.035*	
C15	0.1185 (2)	−0.07129 (14)	1.09604 (13)	0.0273 (4)	
H15	0.0587	−0.0044	1.1291	0.033*	
O2	0.49919 (16)	0.78935 (10)	0.64738 (10)	0.0386 (3)	
H2A	0.4532	0.8172	0.7052	0.046*	
N6	0.84165 (17)	0.33808 (12)	0.64741 (11)	0.0284 (3)	
N7	0.79776 (16)	0.47398 (11)	0.39594 (11)	0.0261 (3)	
N8	0.56651 (17)	0.67789 (12)	0.67161 (12)	0.0315 (3)	
C16	0.94172 (19)	0.24537 (13)	0.47988 (13)	0.0257 (4)	
C17	1.0867 (2)	0.18970 (15)	0.50007 (14)	0.0334 (4)	
H17	1.1349	0.2169	0.5513	0.040*	
C18	1.1611 (2)	0.09363 (17)	0.44459 (15)	0.0391 (5)	
H18	1.2611	0.0546	0.4578	0.047*	
C19	1.0912 (2)	0.05402 (16)	0.37017 (15)	0.0362 (4)	
H19	1.1428	−0.0123	0.3327	0.043*	
C20	0.9459 (2)	0.11116 (16)	0.35027 (14)	0.0340 (4)	
H20	0.8981	0.0843	0.2987	0.041*	
C21	0.8700 (2)	0.20730 (15)	0.40525 (13)	0.0302 (4)	
H21	0.7702	0.2466	0.3919	0.036*	
C22	0.78947 (19)	0.44542 (14)	0.50370 (13)	0.0255 (3)	
C23	0.7165 (2)	0.50922 (14)	0.59080 (13)	0.0266 (4)	
C24	0.7529 (2)	0.43701 (14)	0.67857 (13)	0.0284 (4)	
C25	0.7069 (2)	0.45931 (16)	0.79421 (14)	0.0374 (4)	
H25A	0.7502	0.3924	0.8352	0.056*	
H25B	0.5955	0.4784	0.8089	0.056*	
H25C	0.7447	0.5216	0.8142	0.056*	
C26	0.6762 (2)	0.52578 (16)	0.34263 (14)	0.0352 (4)	
H26	0.5721	0.5447	0.3718	0.042*	
C27	0.7302 (2)	0.54509 (17)	0.24161 (15)	0.0400 (5)	
H27	0.6712	0.5800	0.1874	0.048*	
C28	0.8895 (2)	0.5042 (2)	0.23135 (16)	0.0507 (6)	
H28	0.9576	0.5067	0.1689	0.061*	
C29	0.9289 (2)	0.46064 (19)	0.32625 (16)	0.0435 (5)	
H29	1.0294	0.4268	0.3420	0.052*	
C30	0.6353 (2)	0.62612 (14)	0.58742 (14)	0.0304 (4)	
H30	0.6330	0.6651	0.5214	0.036*	

### Atomic displacement parameters (Å^2^)

**Table:**

	*U*^11^	*U*^22^	*U*^33^	*U*^12^	*U*^13^	*U*^23^
O1	0.0406 (7)	0.0345 (7)	0.0262 (6)	−0.0060 (6)	0.0012 (5)	0.0040 (5)
N1	0.0317 (8)	0.0214 (7)	0.0232 (7)	−0.0070 (6)	−0.0033 (6)	−0.0022 (5)
N2	0.0361 (8)	0.0216 (7)	0.0272 (7)	−0.0071 (6)	−0.0033 (6)	−0.0031 (6)
N3	0.0352 (8)	0.0225 (7)	0.0221 (7)	−0.0070 (6)	−0.0049 (6)	−0.0021 (5)
N4	0.0335 (8)	0.0375 (9)	0.0211 (7)	−0.0105 (7)	−0.0022 (6)	−0.0008 (6)
N5	0.0319 (8)	0.0248 (7)	0.0210 (7)	−0.0067 (6)	−0.0034 (6)	0.0009 (5)
C1	0.0248 (8)	0.0224 (8)	0.0262 (8)	−0.0032 (7)	−0.0008 (7)	0.0002 (6)
C2	0.0308 (9)	0.0255 (9)	0.0271 (9)	−0.0056 (7)	−0.0036 (7)	−0.0011 (7)
C3	0.0325 (10)	0.0320 (10)	0.0266 (9)	−0.0020 (8)	−0.0007 (7)	0.0019 (7)
C4	0.0292 (9)	0.0271 (9)	0.0389 (10)	−0.0035 (7)	0.0020 (8)	0.0066 (8)
C5	0.0253 (9)	0.0273 (9)	0.0446 (11)	−0.0074 (7)	−0.0051 (8)	0.0026 (8)
C6	0.0285 (9)	0.0275 (9)	0.0316 (9)	−0.0044 (7)	−0.0063 (7)	−0.0008 (7)
C7	0.0286 (9)	0.0231 (8)	0.0221 (8)	−0.0038 (7)	−0.0059 (6)	−0.0010 (6)
C8	0.0298 (9)	0.0232 (8)	0.0239 (8)	−0.0057 (7)	−0.0063 (7)	0.0008 (6)
C9	0.0351 (9)	0.0237 (9)	0.0260 (9)	−0.0076 (7)	−0.0045 (7)	0.0001 (7)
C10	0.0643 (14)	0.0278 (10)	0.0358 (11)	−0.0178 (9)	0.0049 (9)	−0.0050 (8)
C11	0.0660 (14)	0.0312 (10)	0.0289 (10)	−0.0105 (9)	−0.0182 (9)	−0.0006 (8)
C12	0.100 (2)	0.0328 (11)	0.0399 (12)	−0.0157 (12)	−0.0292 (12)	−0.0059 (9)
C13	0.0635 (14)	0.0210 (9)	0.0393 (11)	−0.0074 (9)	−0.0091 (9)	−0.0023 (8)
C14	0.0363 (10)	0.0240 (9)	0.0266 (9)	−0.0051 (7)	−0.0037 (7)	0.0013 (7)
C15	0.0331 (9)	0.0254 (9)	0.0236 (8)	−0.0060 (7)	−0.0069 (7)	0.0003 (7)
O2	0.0537 (8)	0.0263 (7)	0.0283 (7)	−0.0002 (6)	0.0026 (6)	−0.0024 (5)
N6	0.0396 (8)	0.0255 (7)	0.0213 (7)	−0.0102 (6)	−0.0049 (6)	0.0012 (6)
N7	0.0287 (8)	0.0253 (7)	0.0227 (7)	−0.0046 (6)	−0.0028 (6)	0.0021 (5)
N8	0.0392 (9)	0.0228 (7)	0.0317 (8)	−0.0064 (6)	−0.0045 (6)	−0.0008 (6)
C16	0.0295 (9)	0.0224 (8)	0.0238 (8)	−0.0054 (7)	−0.0012 (7)	0.0010 (6)
C17	0.0337 (10)	0.0336 (10)	0.0328 (10)	−0.0055 (8)	−0.0095 (8)	−0.0031 (8)
C18	0.0331 (10)	0.0384 (11)	0.0403 (11)	0.0039 (8)	−0.0095 (8)	−0.0042 (8)
C19	0.0395 (11)	0.0302 (10)	0.0338 (10)	−0.0001 (8)	−0.0011 (8)	−0.0070 (8)
C20	0.0373 (10)	0.0365 (10)	0.0281 (9)	−0.0086 (8)	−0.0033 (8)	−0.0079 (8)
C21	0.0270 (9)	0.0333 (10)	0.0294 (9)	−0.0048 (8)	−0.0049 (7)	−0.0028 (7)
C22	0.0273 (9)	0.0241 (8)	0.0249 (8)	−0.0067 (7)	−0.0033 (7)	0.0021 (6)
C23	0.0318 (9)	0.0236 (8)	0.0254 (8)	−0.0091 (7)	−0.0027 (7)	−0.0010 (7)
C24	0.0377 (10)	0.0235 (8)	0.0255 (9)	−0.0108 (7)	−0.0037 (7)	−0.0010 (7)
C25	0.0563 (13)	0.0294 (10)	0.0258 (9)	−0.0107 (9)	−0.0026 (8)	−0.0015 (7)
C26	0.0284 (9)	0.0405 (11)	0.0315 (10)	−0.0001 (8)	−0.0042 (7)	0.0047 (8)
C27	0.0421 (11)	0.0460 (12)	0.0281 (10)	−0.0041 (9)	−0.0072 (8)	0.0061 (8)
C28	0.0393 (12)	0.0726 (16)	0.0317 (11)	−0.0056 (11)	0.0043 (9)	0.0150 (10)
C29	0.0269 (10)	0.0621 (14)	0.0364 (11)	−0.0069 (9)	−0.0001 (8)	0.0143 (9)
C30	0.0385 (10)	0.0261 (9)	0.0256 (9)	−0.0068 (8)	−0.0029 (7)	−0.0004 (7)

### Geometric parameters (Å, º)

**Table:**

O1—N4	1.4078 (18)	C13—H13	0.9500
O1—H1A	0.8403	C14—H14	0.9500
N1—C7	1.358 (2)	C15—H15	0.9500
N1—N2	1.3695 (18)	O2—N8	1.3973 (18)
N1—C1	1.426 (2)	O2—H2A	0.8402
N2—C9	1.330 (2)	N6—C24	1.329 (2)
N3—C14	1.381 (2)	N7—C29	1.376 (2)
N3—C11	1.383 (2)	N7—C26	1.376 (2)
N3—C7	1.397 (2)	N7—C22	1.399 (2)
N4—C15	1.284 (2)	N8—C30	1.280 (2)
N5—C22	1.353 (2)	C16—C17	1.378 (2)
N5—N6	1.3713 (18)	C16—C21	1.384 (2)
N5—C16	1.434 (2)	C17—C18	1.383 (3)
C1—C2	1.386 (2)	C17—H17	0.9500
C1—C6	1.391 (2)	C18—C19	1.380 (3)
C2—C3	1.387 (2)	C18—H18	0.9500
C2—H2	0.9500	C19—C20	1.385 (3)
C3—C4	1.380 (3)	C19—H19	0.9500
C3—H3	0.9500	C20—C21	1.384 (2)
C4—C5	1.382 (3)	C20—H20	0.9500
C4—H4	0.9500	C21—H21	0.9500
C5—C6	1.389 (2)	C22—C23	1.385 (2)
C5—H5	0.9500	C23—C24	1.418 (2)
C6—H6	0.9500	C23—C30	1.449 (2)
C7—C8	1.379 (2)	C24—C25	1.496 (2)
C8—C9	1.421 (2)	C25—H25A	0.9800
C8—C15	1.454 (2)	C25—H25B	0.9800
C9—C10	1.493 (2)	C25—H25C	0.9800
C10—H10A	0.9800	C26—C27	1.351 (3)
C10—H10B	0.9800	C26—H26	0.9500
C10—H10C	0.9800	C27—C28	1.405 (3)
C11—C12	1.345 (3)	C27—H27	0.9500
C11—H11	0.9500	C28—C29	1.353 (3)
C12—C13	1.417 (3)	C28—H28	0.9500
C12—H12	0.9500	C29—H29	0.9500
C13—C14	1.352 (2)	C30—H30	0.9500
			
N4—O1—H1A	101.0	N4—C15—C8	120.32 (16)
C7—N1—N2	110.52 (13)	N4—C15—H15	119.8
C7—N1—C1	130.19 (14)	C8—C15—H15	119.8
N2—N1—C1	119.25 (13)	N8—O2—H2A	105.0
C9—N2—N1	106.20 (13)	C24—N6—N5	105.80 (13)
C14—N3—C11	108.69 (14)	C29—N7—C26	108.17 (15)
C14—N3—C7	127.03 (14)	C29—N7—C22	126.09 (15)
C11—N3—C7	123.98 (15)	C26—N7—C22	125.65 (15)
C15—N4—O1	109.56 (14)	C30—N8—O2	110.12 (14)
C22—N5—N6	110.63 (13)	C17—C16—C21	121.33 (16)
C22—N5—C16	129.45 (14)	C17—C16—N5	119.32 (15)
N6—N5—C16	119.70 (13)	C21—C16—N5	119.33 (15)
C2—C1—C6	120.97 (15)	C16—C17—C18	118.98 (17)
C2—C1—N1	118.81 (15)	C16—C17—H17	120.5
C6—C1—N1	120.18 (15)	C18—C17—H17	120.5
C1—C2—C3	119.25 (16)	C19—C18—C17	120.55 (18)
C1—C2—H2	120.4	C19—C18—H18	119.7
C3—C2—H2	120.4	C17—C18—H18	119.7
C4—C3—C2	120.24 (17)	C18—C19—C20	119.84 (17)
C4—C3—H3	119.9	C18—C19—H19	120.1
C2—C3—H3	119.9	C20—C19—H19	120.1
C3—C4—C5	120.27 (16)	C21—C20—C19	120.25 (17)
C3—C4—H4	119.9	C21—C20—H20	119.9
C5—C4—H4	119.9	C19—C20—H20	119.9
C4—C5—C6	120.36 (17)	C16—C21—C20	119.05 (16)
C4—C5—H5	119.8	C16—C21—H21	120.5
C6—C5—H5	119.8	C20—C21—H21	120.5
C5—C6—C1	118.88 (16)	N5—C22—C23	108.17 (14)
C5—C6—H6	120.6	N5—C22—N7	121.95 (15)
C1—C6—H6	120.6	C23—C22—N7	129.85 (15)
N1—C7—C8	107.93 (14)	C22—C23—C24	104.11 (15)
N1—C7—N3	122.85 (15)	C22—C23—C30	125.38 (15)
C8—C7—N3	129.20 (15)	C24—C23—C30	130.28 (16)
C7—C8—C9	104.66 (15)	N6—C24—C23	111.28 (15)
C7—C8—C15	125.06 (15)	N6—C24—C25	119.72 (15)
C9—C8—C15	130.16 (15)	C23—C24—C25	129.00 (16)
N2—C9—C8	110.67 (15)	C24—C25—H25A	109.5
N2—C9—C10	120.20 (15)	C24—C25—H25B	109.5
C8—C9—C10	129.11 (16)	H25A—C25—H25B	109.5
C9—C10—H10A	109.5	C24—C25—H25C	109.5
C9—C10—H10B	109.5	H25A—C25—H25C	109.5
H10A—C10—H10B	109.5	H25B—C25—H25C	109.5
C9—C10—H10C	109.5	C27—C26—N7	108.32 (16)
H10A—C10—H10C	109.5	C27—C26—H26	125.8
H10B—C10—H10C	109.5	N7—C26—H26	125.8
C12—C11—N3	107.90 (17)	C26—C27—C28	107.58 (17)
C12—C11—H11	126.1	C26—C27—H27	126.2
N3—C11—H11	126.1	C28—C27—H27	126.2
C11—C12—C13	107.91 (17)	C29—C28—C27	107.87 (18)
C11—C12—H12	126.0	C29—C28—H28	126.1
C13—C12—H12	126.0	C27—C28—H28	126.1
C14—C13—C12	107.91 (17)	C28—C29—N7	108.05 (17)
C14—C13—H13	126.0	C28—C29—H29	126.0
C12—C13—H13	126.0	N7—C29—H29	126.0
C13—C14—N3	107.60 (16)	N8—C30—C23	121.29 (16)
C13—C14—H14	126.2	N8—C30—H30	119.4
N3—C14—H14	126.2	C23—C30—H30	119.4
			
C7—N1—N2—C9	−1.45 (18)	C22—N5—N6—C24	1.11 (18)
C1—N1—N2—C9	176.40 (14)	C16—N5—N6—C24	−173.96 (14)
C7—N1—C1—C2	−139.98 (18)	C22—N5—C16—C17	130.07 (19)
N2—N1—C1—C2	42.7 (2)	N6—N5—C16—C17	−55.9 (2)
C7—N1—C1—C6	42.0 (3)	C22—N5—C16—C21	−51.1 (2)
N2—N1—C1—C6	−135.32 (16)	N6—N5—C16—C21	122.89 (17)
C6—C1—C2—C3	0.8 (3)	C21—C16—C17—C18	−0.3 (3)
N1—C1—C2—C3	−177.12 (15)	N5—C16—C17—C18	178.50 (16)
C1—C2—C3—C4	−1.0 (3)	C16—C17—C18—C19	0.0 (3)
C2—C3—C4—C5	0.0 (3)	C17—C18—C19—C20	0.4 (3)
C3—C4—C5—C6	1.2 (3)	C18—C19—C20—C21	−0.5 (3)
C4—C5—C6—C1	−1.4 (3)	C17—C16—C21—C20	0.2 (3)
C2—C1—C6—C5	0.4 (3)	N5—C16—C21—C20	−178.57 (16)
N1—C1—C6—C5	178.29 (15)	C19—C20—C21—C16	0.2 (3)
N2—N1—C7—C8	1.27 (19)	N6—N5—C22—C23	−0.78 (19)
C1—N1—C7—C8	−176.28 (16)	C16—N5—C22—C23	173.68 (16)
N2—N1—C7—N3	179.80 (14)	N6—N5—C22—N7	177.44 (14)
C1—N1—C7—N3	2.3 (3)	C16—N5—C22—N7	−8.1 (3)
C14—N3—C7—N1	49.0 (2)	C29—N7—C22—N5	−50.5 (3)
C11—N3—C7—N1	−124.05 (19)	C26—N7—C22—N5	133.17 (18)
C14—N3—C7—C8	−132.8 (2)	C29—N7—C22—C23	127.3 (2)
C11—N3—C7—C8	54.1 (3)	C26—N7—C22—C23	−49.0 (3)
N1—C7—C8—C9	−0.57 (18)	N5—C22—C23—C24	0.14 (19)
N3—C7—C8—C9	−178.98 (16)	N7—C22—C23—C24	−177.89 (17)
N1—C7—C8—C15	−176.83 (15)	N5—C22—C23—C30	175.12 (16)
N3—C7—C8—C15	4.8 (3)	N7—C22—C23—C30	−2.9 (3)
N1—N2—C9—C8	1.07 (19)	N5—N6—C24—C23	−1.01 (19)
N1—N2—C9—C10	179.60 (16)	N5—N6—C24—C25	179.69 (15)
C7—C8—C9—N2	−0.32 (19)	C22—C23—C24—N6	0.6 (2)
C15—C8—C9—N2	175.67 (16)	C30—C23—C24—N6	−174.07 (17)
C7—C8—C9—C10	−178.69 (19)	C22—C23—C24—C25	179.77 (18)
C15—C8—C9—C10	−2.7 (3)	C30—C23—C24—C25	5.1 (3)
C14—N3—C11—C12	0.1 (2)	C29—N7—C26—C27	−0.3 (2)
C7—N3—C11—C12	174.20 (19)	C22—N7—C26—C27	176.57 (17)
N3—C11—C12—C13	0.0 (3)	N7—C26—C27—C28	0.1 (2)
C11—C12—C13—C14	0.0 (3)	C26—C27—C28—C29	0.2 (3)
C12—C13—C14—N3	0.0 (2)	C27—C28—C29—N7	−0.4 (3)
C11—N3—C14—C13	−0.1 (2)	C26—N7—C29—C28	0.5 (2)
C7—N3—C14—C13	−173.97 (17)	C22—N7—C29—C28	−176.44 (18)
O1—N4—C15—C8	−176.70 (14)	O2—N8—C30—C23	179.19 (15)
C7—C8—C15—N4	−160.65 (17)	C22—C23—C30—N8	177.58 (17)
C9—C8—C15—N4	24.1 (3)	C24—C23—C30—N8	−8.8 (3)

### Hydrogen-bond geometry (Å, º)

*Cg* is centroid of C1–C6 ring.

**Table:**

*D*—H···*A*	*D*—H	H···*A*	*D*···*A*	*D*—H···*A*
O2—H2*A*···N2^i^	0.84	1.95	2.7835 (19)	174
O1—H1*A*···N6^ii^	0.84	1.99	2.8277 (19)	172
C11—H11···*Cg*^ii^	0.95	3.45	?	170

Symmetry codes: (i) *x*, *y*+1, *z*; (ii) −*x*+1, −*y*, −*z*+2.
