# Supplementary figures and images for: Crystal structure of (E)-N-{[3-methyl-1-phenyl-5-(1H-pyrrol-1-yl)-1H-pyrazol-4-yl]methyl­idene}hydroxyl­amine
Source: Acta Crystallogr Sect E Struct Rep Online. 2014 Oct 31;70(Pt 11):o1216–7. doi: 10.1107/S1600536814023514 (PMC4257281; doi:10.1107/S1600536814023514)

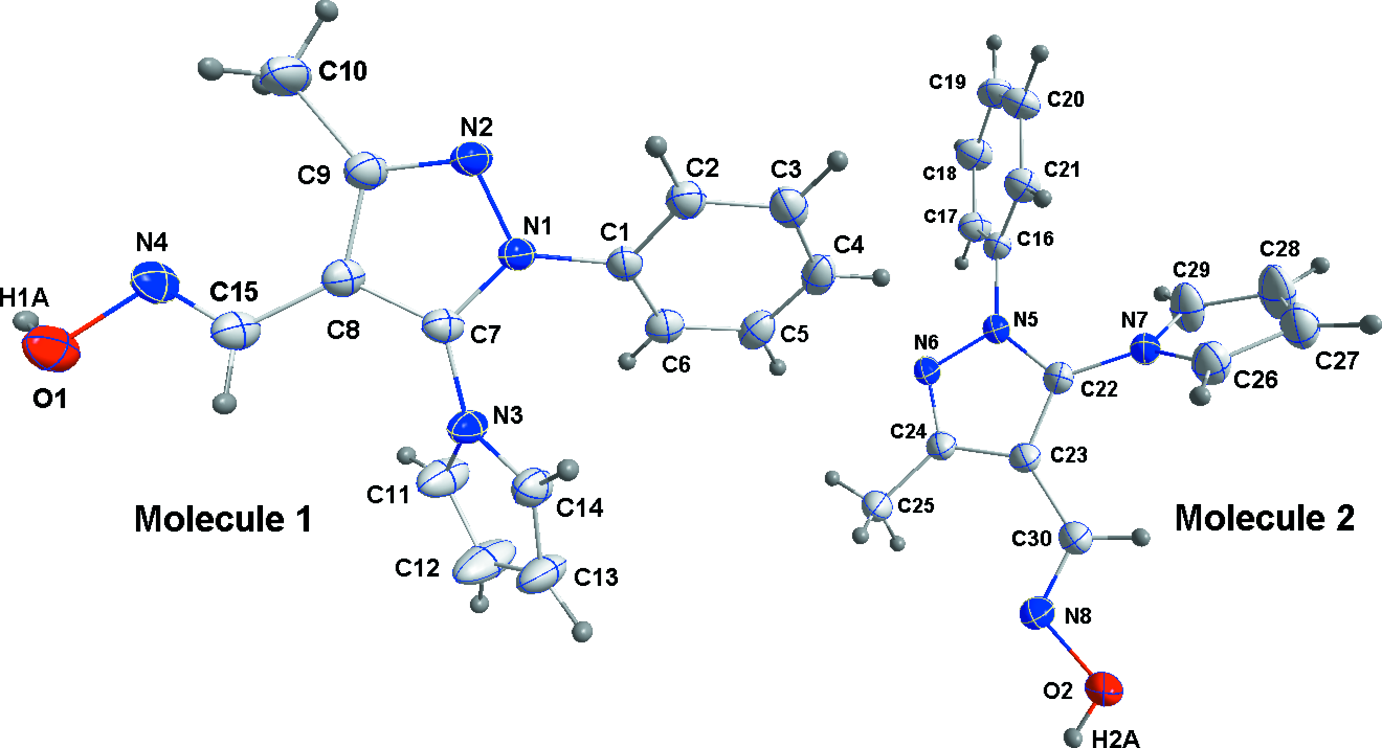

Supplement: Supplementary file 4 [file e-70-o1216-fig1.tif]

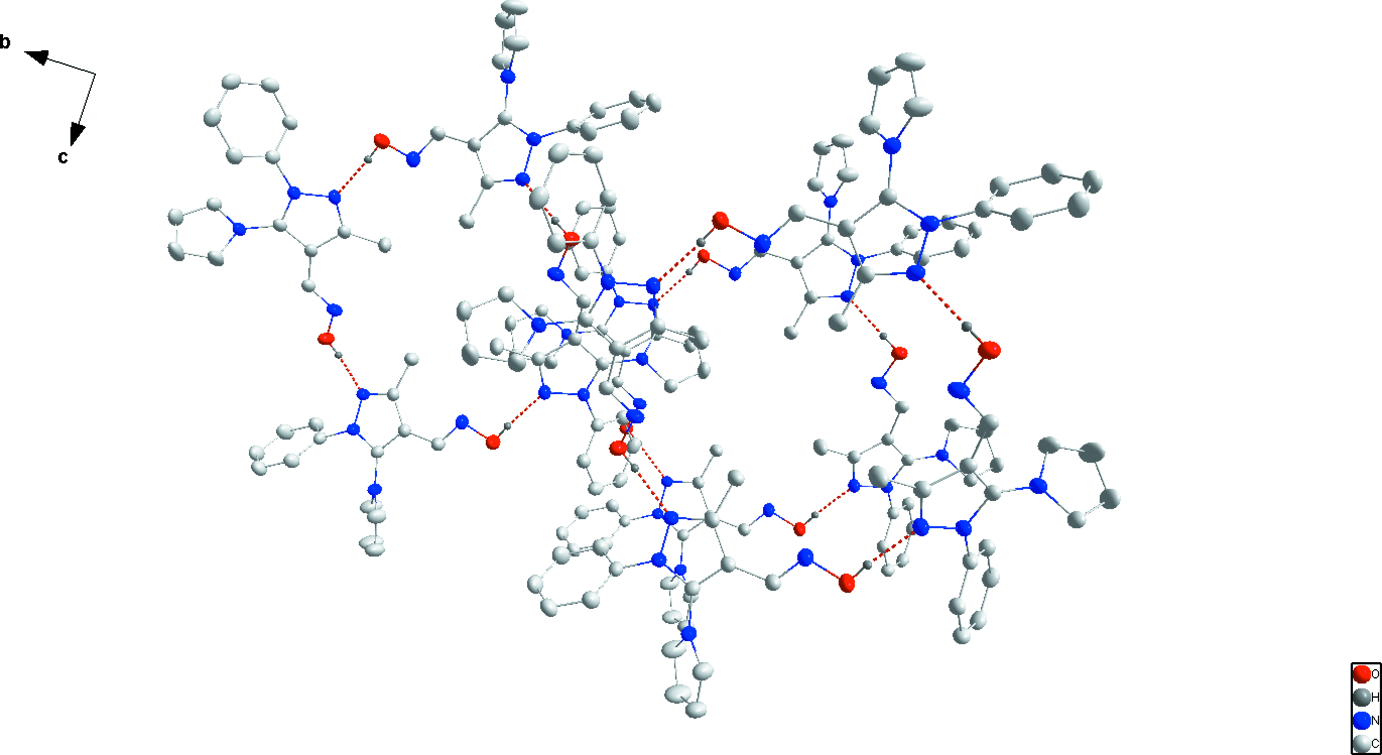

Supplement: Supplementary file 5 [file e-70-o1216-fig2.tif]

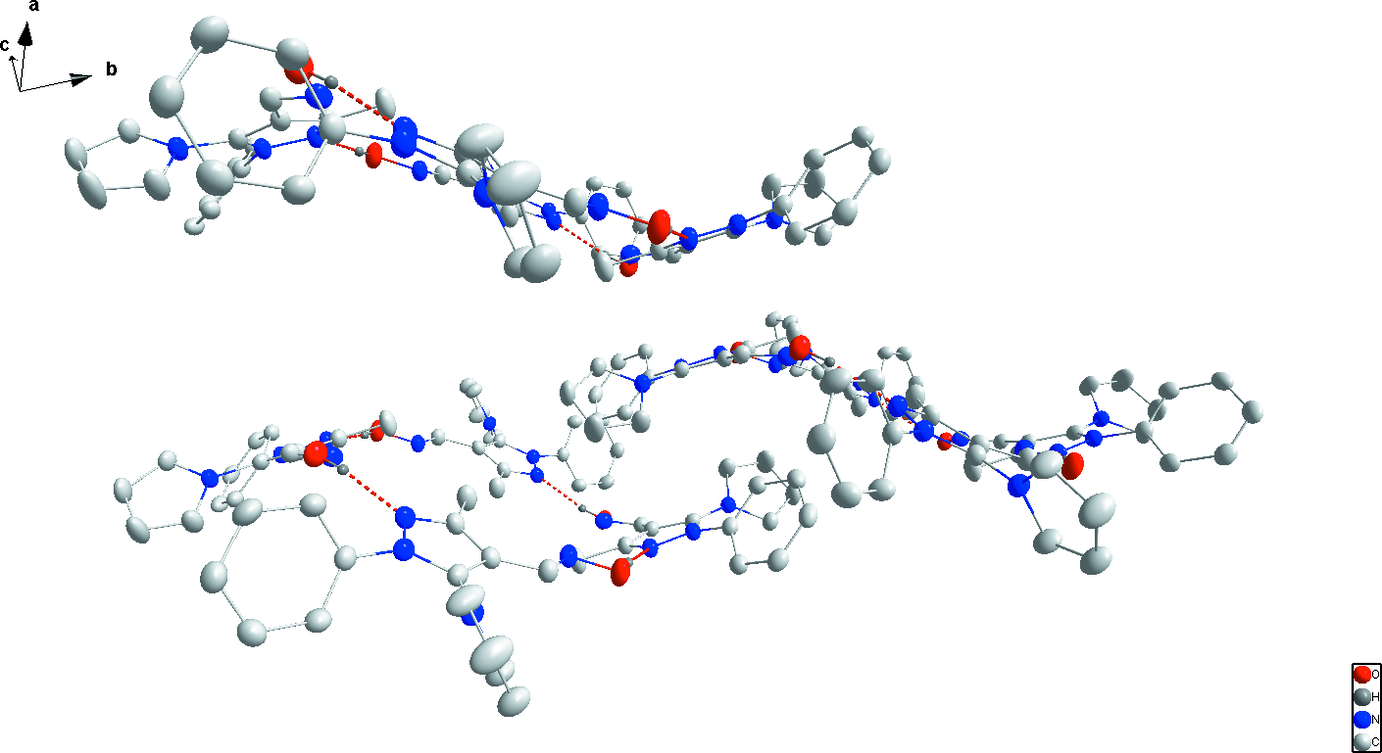

Supplement: Supplementary file 6 [file e-70-o1216-fig3.tif]
